# GMP-grade neural progenitor derivation and differentiation from clinical-grade human embryonic stem cells

**DOI:** 10.1186/s13287-020-01915-0

**Published:** 2020-09-18

**Authors:** Loriana Vitillo, Catherine Durance, Zoe Hewitt, Harry Moore, Austin Smith, Ludovic Vallier

**Affiliations:** 1 Wellcome-MRC Cambridge Stem Cell Institute, Jeffrey Cheah Biomedical Centre; Department of Surgery, University of Cambridge, Cambridge, CB2 0AW UK; 2 The Centre for Stem Cell Biology, Department of Biomedical Science, University of Sheffield, Western Bank, Sheffield, S10 2TN UK; 3 Living Systems Institute, University of Exeter, Exeter, EX4 4QD UK

**Keywords:** Pluripotent stem cells, hESCs, Neural progenitors, GMP, Cell therapy

## Abstract

**Background:**

A major challenge for the clinical use of human pluripotent stem cells is the development of safe, robust and controlled differentiation protocols. Adaptation of research protocols using reagents designated as research-only to those which are suitable for clinical use, often referred to as good manufacturing practice (GMP) reagents, is a crucial and laborious step in the translational pipeline. However, published protocols to assist this process remain very limited.

**Methods:**

We adapted research-grade protocols for the derivation and differentiation of long-term neuroepithelial stem cell progenitors (lt-NES) to GMP-grade reagents and factors suitable for clinical applications. We screened the robustness of the protocol with six clinical-grade hESC lines deposited in the UK Stem Cell Bank.

**Results:**

Here, we present a new GMP-compliant protocol to derive lt-NES, which are multipotent, bankable and karyotypically stable. This protocol resulted in robust and reproducible differentiation of several clinical-grade embryonic stem cells from which we derived lt-NES. Furthermore, GMP-derived lt-NES demonstrated a high neurogenic potential while retaining the ability to be redirected to several neuronal sub-types.

**Conclusions:**

Overall, we report the feasibility of derivation and differentiation of clinical-grade embryonic stem cell lines into lt-NES under GMP-compliant conditions. Our protocols could be used as a flexible tool to speed up translation-to-clinic of pluripotent stem cells for a variety of neurological therapies or regenerative medicine studies.

## Background

The stem cell revolution started with the isolation of human embryonic stem cells (hESCs) [1] followed by the arrival of induced pluripotent stem cells (iPSCs) [2], and their differentiation to an ever-increasing number of cell types has led to the prospect of shifting medicine to a new paradigm based on cellular repair. Despite this enticing prospect, the number of clinical trials based on human pluripotent stem cells (hPSCs) remains limited when compared to other cell types [3]. There is a consensus that hPSCs have a complex and distinct set of scientific, technical and regulatory bottlenecks that hamper their translation to clinical applications [4–7].

One hurdle is that the often-large lists of raw materials used in differentiation protocols are designated of research-grade and were never intended for cell therapy applications. The developers of advanced therapeutic medicinal products (ATMPs) need to follow strict good manufacturing practice (GMP) guidelines to ensure quality and safety of the end products before performing clinical trials. Therefore, raw materials used in differentiation protocols must meet these guidelines. Although policies around raw materials for cell therapeutics are currently flexible, clinical trials regulations require for each reagent to be extensively risk-assessed and qualified [8]. It is in this context that so called GMP-grade materials suitable for clinical use will facilitate clinical trial submission and will likely become standard in the field [9].

To comply with such regulations, hESCs have been derived under clinical-grade conditions [10–12] and GMP-compliant culture platforms have been developed [13, 14]. However, published GMP-compliant differentiation protocols are still notably lacking, but their development would significantly speed the translational pipeline for pluripotent stem cells, particularly for academic groups.

Previous work, including ours, showed that hPSCs could be differentiated into a long-term neuroepithelial-like stem cell population, lt-NES, with stable neurogenic potential towards several neuronal sub-types [15, 16]. In the context of regenerative medicine, a source of neurons that is expandable, bankable and intermediate (i.e. at progenitor stage) has several advantages over run-through protocols. Lt-NES would reduce processing steps, would be a convenient quality control check point and could potentially be used for several applications, facilitate scalability and also by-pass intrinsic line-to-line variability associated with iPSCs [16]. Here, we develop a novel protocol for the derivation and differentiation of lt-NES from clinical-grade hESC lines deposited in the UK Stem Cell Bank based on GMP-grade media and factors.

## Methods

### Cell lines and culture methods

Derivation of the MasterShef-3, -4, -7, -8, -10 and -11 cell lines was performed in the Stem Cell Derivation Facility at the Centre for Stem Cell Biology, University of Sheffield, under HFEA licence R0115-8-A (Centre 0191) and HTA licence 22510, in a clean room setting, following strict standard operating procedures. The embryos used to derive MasterShef-3, -4, -7, 10 (frozen embryos) and MasterShef-8 and -11 (fresh embryos) were donated from different Assisted Conception Units, following fully informed consent, with no financial benefit to the donors, and were surplus or unsuitable for their IVF treatment. Briefly, the embryos were cultured using standard IVF culture media (Medicult), to the blastocyst stage. Following removal of the trophectoderm using a dissection laser, the embryos were explanted whole onto either mitotically inactivated human neonatal fibroblasts (human feeders) in the case of MasterShef-3, -4, -7, -8 and -10 or onto Laminin-511 (Biolamina) in the case of MasterShef11. Derivation media for MasterShef-3, -4, -7 and -8 was standard KSR/KODMEM (Life Technologies) medium while MasterShef-10 and -11 were derived in Nutristem medium (Biological Industries). All cell lines were initially maintained at 37 °C under 5% O_2_/ 5% CO_2_, until the lines were established, after which maintenance switched 5% CO_2_ in air at 37 °C. Cultures were passaged using a manual technique, cutting selected colonies under a dissection microscope at an average split ratio of 1:2 every 7 days. All cell lines have been deposited at the UK Stem Cell bank (https://www.nibsc.org/ukstemcellbank). The H9 cell line was obtained from the WiCell Institute, USA. lt-NES control line, here called AF22, was obtained from A. Smith, Cambridge, UK. AF22 lt-NES were derived from the hiPSCs line AF22 [16].

HESCs were routinely maintained on recombinant VTN-N Vitronectin (A14700, Life Technologies), also tested on the prototype CTS™ (Cell Therapy Systems) Vitronectin with similar results (now A27940, Life Technologies), and GMP Essential 8 (A1517001, Life Technologies). For routine passaging, cells were washed once with CTS™ DPBS^−/−^ (A1285601, Life Technologies) and incubated at room temperature for 1–2 min with GMP EDTA (15,575,020, Invitrogen). After aspiration of EDTA, colonies were gently detached as small clumps and passaged at a ratio of 1:6 without centrifugation. Cells were frozen in animal-free freezing medium, CryoStem (K1-0640, Geneflow), and thawed in presence of GMP ROCK inhibitor Revitacell (1:100, A2644501, Life Technologies).

For traditional research-grade differentiation and reagents, refer to the supplemental experimental procedures.

### Establishment of GMP lt-NES

Undifferentiated hESCs were dissociated into single cells with StemPro Accutase (A1110501, Life Technologies) for 2–3 min at 37 °C, suspended into GMP Essential 6 (A1516401, Life Technologies), counted with a haemocytometer and centrifuged at 300×*g* for 5 min. Cells were suspended at a concentration of 3 × 10^6^ into 1.5 ml of (E6) plus Revitacell, and gently mixed into one well of Aggrewell 800 (Stem Cell Technologies) previously centrifuged at 2000×*g* with 500 μl of E6 plus Revitacell. Embryoid bodies (EBs) formed after 24 h and the media was carefully and completely replaced with fresh E6. From day 2 till day 4 EBs were fed daily with half media change within the Aggrewell. At day 5, EBs were detached from the Aggrewell using a p1000 tip, while a large bore tip (Starlab, E1011–9618) was used for careful collection and deposition of the EBs on the top of a 37-μm reversible strainer (Stem Cell Technologies). Multiple cycles were performed with E6 until all the EBs were removed. EBs were then plated onto 1 well of a 6-well plate (Corning) coated overnight with 10 μg/ml of xeno-free human recombinant Laminin 521 (LN521, Biolamina) prepared in GMP DPBS^+/+^ (A1285801, Life Technologies) by reversing the strainer and washing the EBs into the plate with GMP N2 media (CTS™ DMEM-F12, A1370801; CTS™ N2 1:100, A1370701; CTS™ B27 1:1000, A1486701; 1% GMP Glutamax, A12860-01; Life Technologies). Neural induction was induced for 3–5 days by changing GMP N2 media daily. Neural rosettes were derived between day 3 and 5 by addition of STEMdiff™ Neural Rosette Selection Reagent (05832, Stem Cell Technologies) for 45 min–1 h at 37 °C. The rosettes were gently detached with N2 media directed with a p1000 tip on the visible rosette clusters. Purity of selection was checked under the microscope for detachment of rosette clusters and non-differentiated cells. Removed rosettes were collected in a tube and new media was used to continue selection until 70% of the rosettes were collected. Rosettes were centrifuged at 300×*g* for 5 min and suspended into 400 μl of N2 media plus 10 ng/ml of GMP FGF and GMP EGF (233-GMP-025, 236-GMP-01 M; Bio-techne), named N2 EF media, plus Revitacell. Cells were plated into one to 4 wells of a 48-well plated pre-coated with 10 μg/ml Laminin 521 avoiding over pipetting and formation of single cells. For critical steps and troubleshooting, successful derivation of lt-NES depends on proper attachment. It is recommended to prepare several laminin plates of various sizes during early derivation in order to have reserve plates readily available in the event that attachment is not optimal. A high cell density is required for lt-NES to survive and proliferate, around 70%.

Cells were fed daily with GMP N2 EF media. Once confluent, lt-NES were dissociated with accutase for 1 min at 37 °C, collected by pipetting on the surface and suspended into 10 ml N2 media prior to centrifugation at 300×*g* for 5 min. Cells were passaged at a split ratio of 1:1 from a 48-well format to a 6-well plate with addition of Revitacell for the first 24 h. Once cells were in 6-well format, Revitacell was not used during passaging and lt-NES were split at a ratio of 1:2 or 1:3.

### GMP lt-NES maintenance

lt-NES were routinely cultured in GMP N2 EF media on 10 μg/ml Laminin 521. Cells were split every 3–4 days when sub-confluent with incubation with Accutase 1–2 min at 37 °C (without waiting for the cells to be floating in the media) and suspended into 10 ml N2 media before centrifugation at 300×*g* for 5 min. Cells were frozen with CryoStem. lt-NES were thawed at 37 °C for 2 min and immediately resuspended into 10 ml N2 media, centrifuged at 300×*g* for 5 min and plated in N2 EF media plus Revitacell for the first 24 h.

### Spontaneous differentiation of lt-NES

lt-NES were plated at a density of 40,000 cells/cm^2^ on Laminin 521-coated plates in N2 media plus Revitacell for 24 h. The next day, media was changed to terminal differentiation media composed of 50:50 parts of CTS™ DMEM-F12 (with CTS™ N2 1:100) and CTS™ Neurobasal (A1371201, Life Technologies) (with CTS™ B27 1:50) media plus 300 ng/ml cAMP (Sigma Aldrich). Spontaneous differentiation was induced with the media above for 21 continuous days.

### Directed GMP differentiation into dopaminergic neurons

lt-NES were plated at a density of 40,000 cells/cm^2^ on Laminin 521-coated plates in N2 media plus Revitacell for 24 h. The next day, media was changed to dopaminergic patterning medium composed of CTS™ DMEM-F12 (with CTS™ N2 1:100) plus freshly added 200 ng/ml GMP Sonic Hedgehog (SHH, 130-095-727, Miltenyi Biotec), 100 ng/ml GMP FGF-8b (130–095-740, Miltenyi Biotec) and 160 μM Ascorbic Acid (95210-250G, Sigma Aldrich). Cells were cultured in dopaminergic patterning medium for 2 weeks. On day 14, media was changed into terminal differentiation medium composed of equal parts of CTS™ DMEM-F12 (CTS™ N2 1:100) to CTS™ Neurobasal (CTS™ B27 1:50) plus 20 ng/ml GMP BDNF (248-GMP-025, Bio-techne), 10 ng/ml GMP GDNF (212-GMP-050, Bio-techne), 160 μM Ascorbic Acid (Sigma Aldrich) and 500 μM dy-cAMP (Sigma Aldrich). Cells were continuously fed with terminal differentiation media until day 21, when neurons are ready for immunofluorescence characterisation.

### Directed GMP differentiation into motoneurons

lt-NES were plated at a density of 40,000 cells/cm^2^ on Laminin 521-coated plates in N2 media plus Revitacell for 24 h. The next day, media was changed to motoneuron patterning medium composed of CTS™ DMEM F12 (With CTS™ N2 1:100, CTS™ B17 1:50) plus 10 ng/ml GMP EGF (Bio-techne), 10 ng/ml GMP FGF (Bio-techne) and 1 μM retinoid acid (Sigma Aldrich). On day 5, the above media was supplemented with 1 μg/ml GMP SHH (Bio-techne). From day 7, the concentration of retinoid acid was reduced down to 0.01 μM and EGF and FGF were completely removed. On day 12, media was changed to terminal differentiation media composed of equal parts of CTS™ DMEM-F12 (CTS™ N2 1:100) to CTS™ Neurobasal (CTS™ B27 1:50) plus 20 ng/ml GMP BDNF (Bio-techne), 20 ng/ml GMP GDNF (Bio-techne), 50 ng/ml SHH (Bio-techne) and 300 ng/ml cAMP (Sigma Aldrich).

## Results

### Development of an efficient GMP-compatible protocol for lt-NES derivation

In order to develop a GMP-compatible protocol for lt-NES derivation, we used H9 hESCs routinely cultured on a widely recognised and defined culture system based on recombinant vitronectin and Essential 8 (E8) [13]. We started by examining the performance of an embryoid body (EB)-based neural differentiation system under standard research-grade [16] versus GMP media conditions. HESCs were allowed to aggregate spontaneously in suspension for 5 days in research-grade knockout serum replacement differentiation media (KSR) as previously described [16] or in GMP-grade essential 6 (E6), which is composed similarly to GMP E8 but without bFGF or TGFβ and thus is suitable for differentiation. The hESCs formed compacted and round-shaped EBs in KSR, while in E6 they showed an elevated level of attachment to the ultra-low adherence dish and disaggregated into smaller pieces over the 5-day period (Fig. S1A). Consequently, we observed that poorly formed EBs in E6 were also inefficient during neural induction, assessed by the hallmark of neural rosettes, compared to those in KSR (Fig. S1 B).

**Fig. 1:**
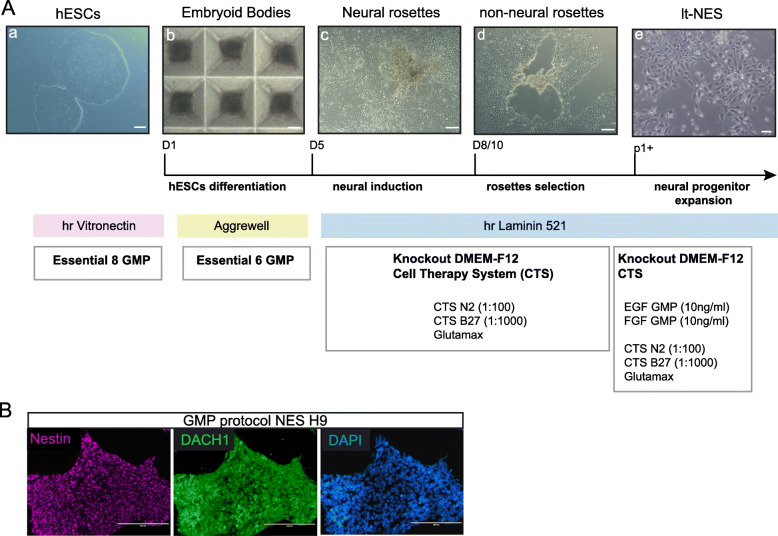
Development of an efficient GMP-compatible protocol for lt-NES. **a** Step-by-step diagram of GMP-compatible differentiation protocol of hESCs into lt-NES. Scale bars (a, c, d) 100 μm, (b) 50 μm and (e) 20 μm. **b** Immunofluorescence for lt-NES markers Nestin and Dach1 in research-grade line H9 after derivation with GMP-compatible protocol. Scale bars 200 μm

Moreover, although neural differentiation is clearly possible via classic spontaneous EB formation methods, this system is not standardised, as the size and the shape of EBs is uncontrolled and this impacts on the reproducibility of differentiation and yield. Therefore, we next examined the performance of an alternative method to produce EBs of defined size based on seeding dissociated cells in microwells. HESCs were dissociated into single cells and seeded at a concentration of 10,000 hESCs per microwell in the presence of a GMP-grade ROCK inhibitor (Revitacell, Life Technologies) in either E6 or KSR. After 24 h, similar-sized EBs were formed in both conditions and at day 5 they remained aggregated (Fig. 1A and S1 C). Upon dissociation, an equal number of same-sized EBs was obtained with this protocol from both KSR and E6 media. We tested the neural induction efficiency of standardised EBs by looking for the emergence of neural rosettes after plating in neuronal inducing conditions. Surprisingly, neural rosettes emerged more prevalently from EBs derived from E6 rather than KSR, in contrast to the spontaneous differentiation system (Fig. S1 C). Moreover, GMP-neural induction was robust and highly efficient, as shown by rosettes forming simultaneously and similarly at the centre of plated EBs within 3 days (Movie 1). We also tested neural induction on a defined laminin matrix, laminin 521, now available in GMP-grade, showing similar results to standard research-grade poly-L-Ornithine/laminin substrate (Fig. S1 D).

Derivation of lt-NES from neural rosettes has previously been described using a manual picking system with needle dissection of rosettes [15, 16]. In the context of future manufacturing applications for lt-NES, we tested the suitability of a commercially available rosette selection solution (STEMdiff™ Neural Rosette Selection, Stem Cell Technologies) with our GMP protocol. Application of the reagent allowed the pipetting out of loosened neural rosette cluster from a surrounding non-neural rosette ring of differentiated cells without the need for manual dissection (Fig. 1A). With this method, lt-NES were derived after disaggregating the rosettes and plating the cells at high density in the presence of GMP-grade EGF and FGF and cultured thereafter until sub-confluent. Under these culture conditions, it-NES could be expanded every 2–3 days, a result of their typical self-renewing capacity, while maintaining a highly pure population (Fig. 1A). Indeed, characteristic lt-NES markers Nestin and Dach1 were expressed homogeneously by the cultures differentiated with this protocol (Fig. 1B).

We concluded that the simultaneous combination of GMP-grade reagents (Table 1), standardised EB formation and regent-based rosette isolation provides an efficient system for the derivation of lt-NES using a GMP-compatible platform.

### Neural differentiation potential of clinical-grade hESC lines

**Fig. 2:**
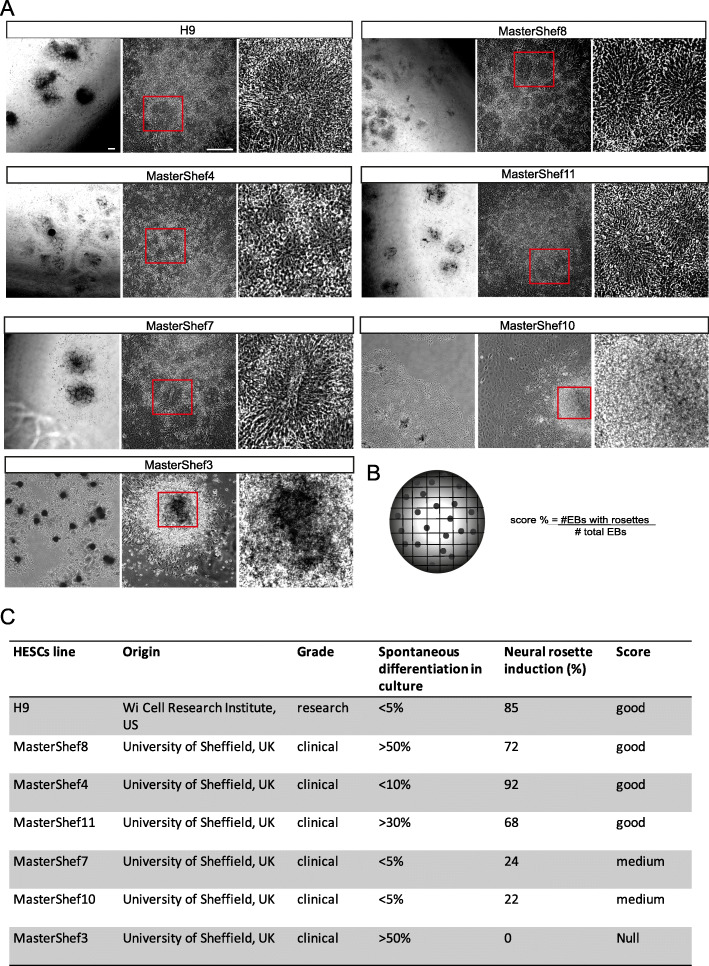
Neural differentiation potential of clinical grade hESC lines. **a** Representative phase contrast images of H9 and clinical-grade hESCs differentiated under GMP-compliant protocol into neural rosettes. Enlarged neural rosettes are visible in the right-hand side panels. Scale bars 200 μm. **b** Formula for the calculation of neural induction efficiency based on neural rosette in hESC lines. **c** Summary of screening of research and clinical-grade hESCs for neural induction capacity under GMP-compatible protocol

With a new GMP-compatible lt-NES derivation protocol developed, we next examined its robustness by screening a panel of 6 clinically derived hESC (MasterShef) lines. MasterShef-3, -4, -7, -8, -10 and -11 were derived at, and obtained from, the Centre for Stem Cell Biology, University of Sheffield, under a HTA licence for potential clinical application (22510). With this approach, we aimed to also examine the specific neural differentiation potential of these clinical-grade lines which have been deposited in the UK Stem Cell Bank. Since for the derivation of lt-NES it is essential to generate neural rosettes, we decided to assess the differentiation potential based on the ability of the lines to give rise to morphologically distinct neural rosettes. The same number of cells for each line was differentiated into neural rosettes following our protocol and rosette formation was recorded by imaging the whole cell culture vessel with high definition imaging using Biostation CT twice daily for 5 days following EB plating (Fig. 2a). Four out of six MasterShef lines [4, 7, 8, 11], as well as an additional hESC research line from a different source, H9, were able to differentiate into neural rosettes, showing that the protocol is robust across several different lines (Fig. 2a). Next, we scored each line for the percentage of neural induction by counting the numbers of EBs hosting neural rosettes in the whole-vessel images at the end of the induction, normalised to the total number of EBs attached (Fig. 2b). The efficiency of neural induction is summarised in Fig. 2c. We defined ‘good’ scores when more than 50% of the EBs carried rosettes, ‘medium’ scores when the value was below 50% but above 5%, and null when rosettes were undetected. Good neural induction scores were obtained regardless of the general level of spontaneous differentiation in pluripotency maintenance conditions assessed by daily morphological monitoring of the cultures for signs of differentiation (Fig. 2c).

Overall, these data demonstrate that our GMP-compatible protocol is suitable for an efficient neural differentiation of several clinically relevant hESCs without the need for cell line-specific optimisation.

### Establishment and characterisation of GMP-compatible lt-NES

**Fig. 3:**
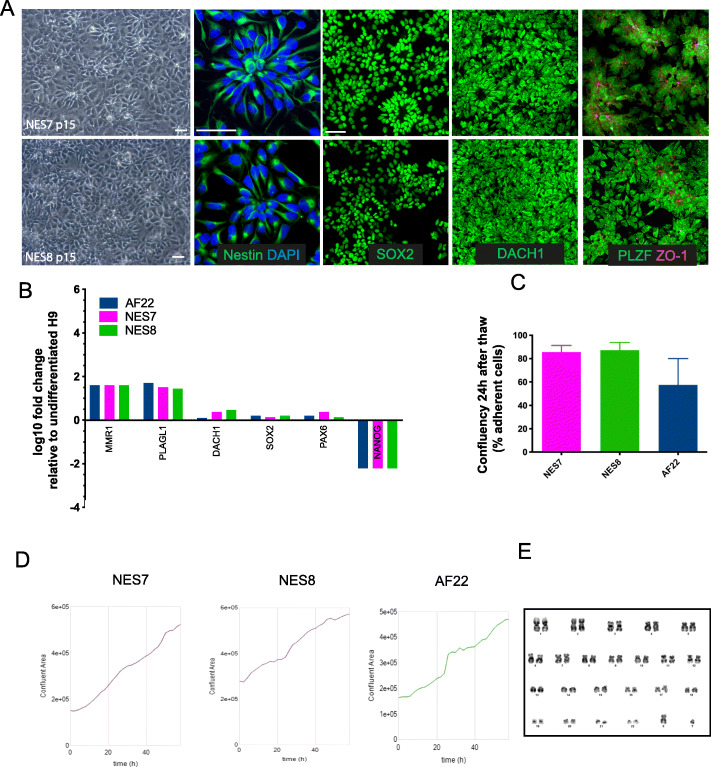
Establishment and characterisation of GMP-compatible lt-NES. **a** Representative phase contrast showing morphology of lt-NES derived from clinical grade MasterShef 7 (NES7) and 8 (NES8). Scale bars 20 μm. NES7 and NES8 cells were immunostained for the lt-NES markers Nestin, SOX2, Dach1, PLZF and the polarity marker ZO-1. Scale bars 50 μm. **b** Gene expression levels of lt-NES markers in NES7 and NES8 compared to research-grade AF22 lt-NES line and assessed by Q-PCR (*n* = 3). **c** Recovery after thaw of NES cells at 24 h. Graph shown as mean plus SEM (*n* = 3). **d** Representative growth curve of NES7, NES8 and control AF22 cultured under GMP-compatible conditions on L521. **e** Normal karyology of established NES7 at passage 15 cultured in GMP-compatible system

After screening clinical-grade hESCs with our protocol, we examined if bona-fide GMP-compatible lt-NES could be derived and maintained from these lines. We successfully established new lt-NES from both a good score, MasterShef 8 and a medium score line, MasterShef 7, hESC line, which we named NES8 and NES7, respectively. NES7 and NES8 showed typical lt-NES morphology and self-organised in rosette-like clusters (Fig. 3a), similarly to the published research-grade lt-NES AF22 (Fig. S2). Furthermore, they homogeneously expressed lt-NES markers Nestin, SOX2, DACH1, PLZF and the polarity marker ZO-1 by immunofluorescence (Fig. 3a). Consistently, NES7 and NES8 also expressed high level of lt-NES-specific markers by Q-PCR, comparably to control lt-NES AF22 (Fig. 3b). Our cells also preserved particularly useful features of lt-NES in the context of cell therapy manufacturing, such as good recovery after freeze-thaw (Fig. 3c) and exponential proliferation in GMP conditions (Fig. 3d) while retaining a rosette-like morphology (Movie 2). Finally, lt-NES grown on laminin 521 maintained a normal karyotype (results from 30 spreads) after more than 15 passages (Fig. 3e).

These results demonstrate that GMP-compatible lt-NES derived from clinical-grade hESCs are comparable to research-grade lt-NES in morphology, markers and proliferative attributes.

### Spontaneous and directed GMP-compatible differentiation of lt-NES

Lt-NES have been shown to have a spontaneous bias towards hindbrain phenotypes, nevertheless retain multipotency and can be directed to differentiate into other neuronal cell types [15–17]. Therefore, we examined whether our GMP-compatible lt-NES were able to differentiate into neurons in GMP-compatible differentiation conditions, using laminin 521 as substrate.

**Fig. 4:**
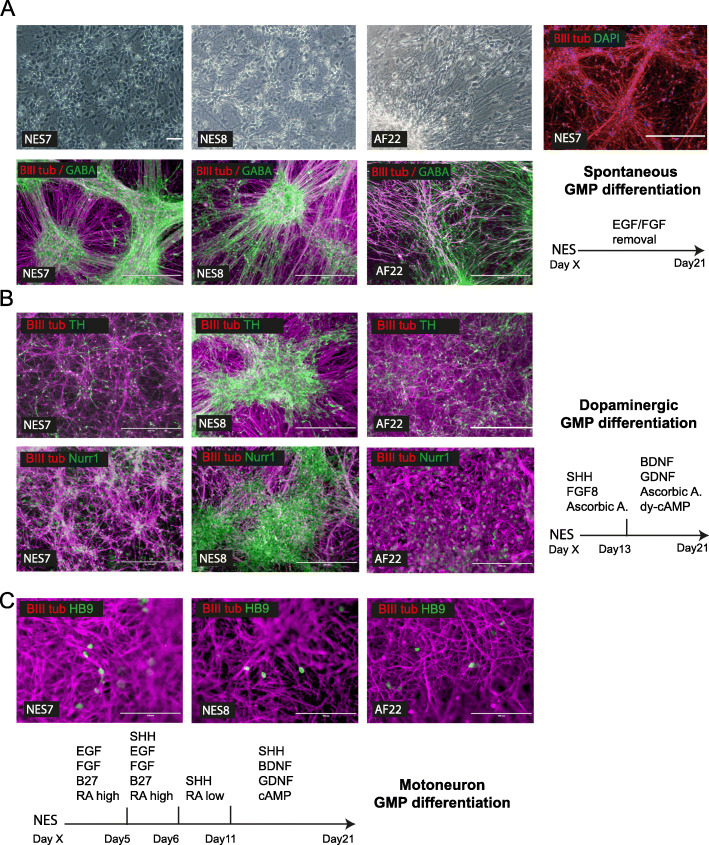
Spontaneous and directed GMP-compatible differentiation of lt-NES. **a** Spontaneous differentiation of NES lines into neurons under GMP-compatible conditions. Spontaneous neurogenic potential shown by phase contrast (scale bars 100 μm) and immunostaining for neuronal marker BIII Tubulin (NES7; scale bar 400 μm). Typical hindbrain bias of lt-NES shown by expression of GABAergic markers GABA (scale bars 400 μm). **b** Directed differentiation of NES lines into midbrain dopaminergic neurons under GMP-compatible conditions. Immunostaining images showing positivity for dopaminergic markers tyrosine hydroxylase (TH) and Nurr1. Scale bars 400 μm (200 μm for AF22 Nurr1). **c** Directed differentiation of NES lines into motoneurons under GMP-compatible conditions showing motor neuron marker HB9 expression by immunofluorescence. Scale bars 100 μm

Firstly, we assessed our lt-NES spontaneous neurogenic potential by removing growth factors and allowing differentiation in neurogenic GMP media for 21 days by adapting research-grade protocols [16]. We observed that both NES7 and NES8 had a high neurogenic potential, giving rise to a homogenous and interlinked network of neurons over the course of the differentiation (Movie 3 and Fig. 4). Indeed, the differentiated lt-NES expressed the neuronal marker Beta III tubulin (Fig. 4a). Moreover, we confirmed the GABAergic propensity of the lt-NES since a large number of neurons were positive for the GABA marker, in line with previous reports [18] and similarly to the control line AF22 (Fig. 4a).

Secondly, we examined the potential of our lt-NES to be redirected to alternative neuronal types under GMP differentiation conditions. Substituting reagents from previous lt-NES research methods [15] with GMP-grade equivalents, we tested differentiation towards a dopaminergic phenotype, which could have interest for cell transplant studies for Parkinson’s disease. Our results show that our GMP lt-NES method was able to give rise to neurons expressing dopaminergic markers tyrosine hydroxylase (TH) and Nurr1 after 21 days (Fig. 4b).

Finally, lt-NES could also be directed towards a motoneuron phenotype in GMP conditions recapitulating research-grade protocols [15], even if much less efficiently than for the dopaminergic differentiation, as showed by detection of the mature motoneuron marker HB9 by immunofluorescence (Fig. 4c).

In conclusion, our data demonstrated the feasibility of a fully defined and GMP-compatible protocol for the derivation and differentiation of hESCs into neurons via stable and expandable intermediate progenitor lt-NES.

## Discussion

In this study, we established a system for the derivation, maintenance and differentiation of neuroepithelial stem cells lt-NES under GMP-compliant conditions suitable for clinical applications. We substituted each reagent of the original research-grade protocols [15, 16] with available reagents of sufficient quality standards to allow their clinical application, so called GMP-approved reagents or cell therapy-grade reagents. Manufacturers of such reagents have either lodged ‘drug master files’ with regulatory authorities or are able to provide the detailed quality documentation required to perform a full risk assessment, which would in turn satisfy an appropriate regulator. In a few circumstances, when such grade was not readily available, we implemented reagents that are fully defined or which are in the process of achieving this standard by the manufacturers.

We also optimised the original differentiation protocol for transition to cell manufacturing environments. We found that a controlled and standardised EB formation was not only desirable but necessary for use of GMP Essential 6 media in the first phase of the protocol. In practice, this led to higher reproducibility and yield of neural induction compared to the traditional KSR system. It would be interesting to investigate across a greater number of differentiation platforms whether the use of GMP-grade reagents provides an avenue for improvement of current research protocols. It would also be interesting to evaluate whether the choice of routine pluripotency maintenance conditions affects downstream differentiation.

A critical aspect of hESCs is their intrinsic line-to-line variability [19]. In the context of cell therapy, our new protocol demonstrated robustness when applied to six different clinical-grade hESC lines with a 66.6% efficiency. Moreover, lt-NES derived from hESCs with different neural differentiation propensities expressed similar characterisation attributes between each other and to a control research-grade line. These findings confirm previous data reporting that lt-NES derivation may circumvent upstream differences between hPSC lines [16], but here also showing comparability across different derivation conditions (i.e. GMP vs research protocol). Indeed, our control NES line used throughout this study, AF22 [16], not only was derived under research-grade protocols but also from a human iPSC line. The ability of hiPSC-derived AF22 to be expanded and terminally differentiated with our GMP protocol in parallel to hESCs-derived lt-NES suggest that the protocol is applicable to both hESC and iPSC-derived lt-NES. Nonetheless, it would be important to test the entire GMP derivation on a set of clinical-grade hiPSCs as they will become increasingly available to the cell therapy community. As expected, a few of the screened lines were not able to differentiate efficiently under this protocol, reflecting the known characteristic of hESC lines to have different developmental potentials. In this regard, this study strengthens the view that screening a panel of pluripotent lines is crucial for cell therapy applications.

We also established that GMP-grade maintenance conditions support the features of self-renewing lt-NES: rosette-like morphology, homogeneous and stable expression of neural rosettes markers, long-term expansion in EGF/FGF, resistance to repeated freeze/thawing and stable karyotype. Moreover, our lt-NES displayed high neurogenic potential towards GABAergic sub-types upon growth factors removal, in line with their hindbrain identity [15, 16]. lt-NES could be successfully re-specified towards adjacent regional cell types such as dopaminergic neurons and to a less degree also to motor neurons under GMP-culture conditions, confirming their multipotentiality. Therefore, with these protocols, we intend to offer a flexible starting point and cut the burden of time-consuming and expensive process development.

The clinical future of pluripotent stem cell-derived therapeutics will likely depend on our ability to tackle several roadblocks associated with the development of cell manufacturing processes, of which adaptation to suitable qualified materials is a crucial phase [4–7]. This study shows that translation of research-grade protocols to GMP-compliant protocols can be effectively achieved and that making these changes can lead to robust and efficient processes. Stem cell researchers looking at transitioning to clinic can take our results as positive evidence that the original research protocol blueprint can be maintained and built upon. However, the time and cost commitment to achieving this should not be under-estimated, and with few reports in the literature to document these processes, many developers have to start from the beginning.

## Conclusion

Overall, the findings of the present report demonstrate feasibility of a GMP-compliant differentiation protocol for intermediate neural progenitors that are easy to expand, bankable and amenable to downstream differentiation into different neuronal sub-types. In the context of the cell therapy field, we report pre-screening of the neuronal differentiation capacity of 6 clinical-grade MasterShef hESC lines deposited in the UK Stem Cell Bank and established a resource in GMP lt-NES that could be used for further optimization depending on the required therapeutic goal. Recently, a method for differentiating lt-NES towards astroglia has been developed, opening new avenues for the downstream therapeutic use of GMP lt-NES [17]. Considering the promising comparability between our GMP lt-NES and the AF22 lt-NES also used by Lundin et al. [17], it would be interesting to test the performance of our GMP lt-NES towards astroglia in future studies. Given its robustness and flexibility, our protocol could be applied for the generation of GMP-compliant neural progenitors that are potentially employable for a variety of neurological therapies, for cell manufacturing scalability studies, drug screenings and other biomedical research applications [20].

## Supplementary information

**Additional file 1: Figure S1.** Development of an efficient GMP-compatible protocol for lt-NES (Related to Fig. 1). **Figure S2.** Establishment and characterisation of GMP-compatible lt-NES (Related to Fig. 3).

**Additional file 2: Table 1**. Regents used in research-grade versus GMP-grade protocols.

**Additional file 3: Movie 1.** Neural rosette formation. (MOV 10158 kb)

**Additional file 4: Movie 2.** Lt-NES proliferation. (MOV 21778 kb)

**Additional file 5: Movie 3.** Lt-NES spontaneous differentiation into neurons. (MOV 142656 kb)

**Additional file 6: Supplemental methods**. Supplemental experimental procedures.

## Data Availability

The datasets used and/or analysed during the current study are available from the corresponding author on reasonable request.

## Abbreviations

EBs: Embryoid bodies
EGF: Epithelial growth factor
E6: Essential 6
E8: Essential 8
FGF: Fibroblast growth factor
GMP: Good manufacturing practice
hESC: Human embryonic stem cell
iPSC: induced pluripotent stem cell
lt-NES: Long-term neuroepithelial stem cells
KSR: Knockout serum replacement

## References

[1] Thomson JA, Itskovitz-Eldor J, Shapiro SS, Waknitz MA, Swiergiel JJ, Marshall VS (1998). Embryonic stem cell lines derived from human blastocysts. Science..

[2] Takahashi K, Yamanaka S (2006). Induction of pluripotent stem cells from mouse embryonic and adult fibroblast cultures by defined factors. Cell..

[3] Trounson A, McDonald C (2015). Stem cell therapies in clinical trials: progress and challenges. Cell Stem Cell.

[4] Williams DJ, Archer R, Archibald P, Bantounas I, Baptista R, Barker R (2016). Comparability: manufacturing, characterization and controls, report of a UK Regenerative Medicine Platform Pluripotent Stem Cell Platform Workshop, Trinity Hall, Cambridge, 14-15 September 2015. Regen Med.

[5] Whiting P, Kerby J, Coffey P, da Cruz L, McKernan R (2015). Progressing a human embryonic stem-cell-based regenerative medicine therapy towards the clinic. Philos Trans R Soc Lond Ser B Biol Sci.

[6] Ratcliffe E, Glen KE, Naing MW, Williams DJ (2013). Current status and perspectives on stem cell-based therapies undergoing clinical trials for regenerative medicine: case studies. Br Med Bull.

[7] Abbasalizadeh S, Baharvand H (2013). Technological progress and challenges towards cGMP manufacturing of human pluripotent stem cells based therapeutic products for allogeneic and autologous cell therapies. Biotechnol Adv.

[8] Stacey G, Andrews P, Asante C, Barbaric I, Barry J, Bisset L (2018). Science-based assessment of source materials for cell-based medicines: report of a stakeholders workshop. Regen Med.

[9] Solomon J, Csontos L, Clarke D, Bonyhadi M, Zylberberg C, McNiece I (2016). Current perspectives on the use of ancillary materials for the manufacture of cellular therapies. Cytotherapy..

[10] Hewitt ZA, Amps KJ, Moore HD (2007). Derivation of GMP raw materials for use in regenerative medicine: hESC-based therapies, progress toward clinical application. Clin Pharmacol Ther.

[11] Jacquet L, Stephenson E, Collins R, Patel H, Trussler J, Al-Bedaery R (2013). Strategy for the creation of clinical grade hESC line banks that HLA-match a target population. EMBO Mol Med.

[12] Wang J, Hao J, Bai D, Gu Q, Han W, Wang L (2015). Generation of clinical-grade human induced pluripotent stem cells in Xeno-free conditions. Stem Cell Res Ther.

[13] Chen G, Gulbranson DR, Hou Z, Bolin JM, Ruotti V, Probasco MD (2011). Chemically defined conditions for human iPSC derivation and culture. Nat Methods.

[14] Nakagawa M, Taniguchi Y, Senda S, Takizawa N, Ichisaka T, Asano K (2014). A novel efficient feeder-free culture system for the derivation of human induced pluripotent stem cells. Sci Rep.

[15] Koch P, Opitz T, Steinbeck JA, Ladewig J, Brustle O (2009). A rosette-type, self-renewing human ES cell-derived neural stem cell with potential for in vitro instruction and synaptic integration. Proc Natl Acad Sci U S A.

[16] Falk A, Koch P, Kesavan J, Takashima Y, Ladewig J, Alexander M (2012). Capture of neuroepithelial-like stem cells from pluripotent stem cells provides a versatile system for in vitro production of human neurons. PLoS One.

[17] Lundin A, Delsing L, Clausen M, Ricchiuto P, Sanchez J, Sabirsh A (2018). et al. Human iPS-derived astroglia from a stable neural precursor state show improved functionality compared with conventional astrocytic models. Stem Cell Reports.

[18] Tailor J, Kittappa R, Leto K, Gates M, Borel M, Paulsen O (2013). Stem cells expanded from the human embryonic hindbrain stably retain regional specification and high neurogenic potency. J Neurosci.

[19] Cahan P, Daley GQ (2013). Origins and implications of pluripotent stem cell variability and heterogeneity. Nat Rev Mol Cell Biol.

[20] Huang M, Tailor J, Zhen Q, Gillmor AH, Miller ML, Weishaupt H (2019). Engineering genetic predisposition in human neuroepithelial stem cells recapitulates medulloblastoma tumorigenesis. Cell Stem Cell.

