# Supplementary material for: GMP-grade neural progenitor derivation and differentiation from clinical-grade human embryonic stem cells
Source: Stem Cell Res Ther. 2020 Sep 18;11:406. doi: 10.1186/s13287-020-01915-0 (PMC7501686; doi:10.1186/s13287-020-01915-0)
Supplement: Supplementary file 1 — Additional file 1: Figure S1. Development of an efficient GMP-compatible protocol for lt-NES (Related to Fig. 1). Figure S2. Establishment and characterisation of GMP-compatible lt-NES (Related to Fig. 3). [file 13287_2020_1915_MOESM1_ESM.docx]

**FIGURE S1**

**Figure S1 Development of an efficient GMP-compatible protocol for lt-NES (Related to Figure 1)**

1. Representative phase images of embryoid bodies spontaneously formed from hESCs (H9) in suspension on low-adherence plates in KSR or Essential 6 media.
2. Representative phase images of neural rosettes induction from spontaneously formed EBs (from H9) in KSR versus Essential 6 media.
3. Representative comparison of standardized neural rosette induction (from MShef10) under research-grade KSR or GMP Essential 6 media.
4. Representative phase images of neural rosettes formation (from H9) on research-grade substrate made of poly-L-Ornithine/laminin versus the defined recombinant laminin 521 matrix.

Scale bars 100 μm

**FIGURE S2**

**Figure S2 Establishment and characterization of GMP-compatible lt-NES**

**(Related to Figure 3)**

1. Representative phase image of the established research-grade lt-NES line AF22. Scale bar 100 μm
