# Supplementary material for: GMP-grade neural progenitor derivation and differentiation from clinical-grade human embryonic stem cells
Source: Stem Cell Res Ther. 2020 Sep 18;11:406. doi: 10.1186/s13287-020-01915-0 (PMC7501686; doi:10.1186/s13287-020-01915-0)
Supplement: Supplementary file 6 — Additional file 6: Supplemental methods. Supplemental experimental procedures. [file 13287_2020_1915_MOESM6_ESM.docx]

**SUPPLEMENTAL EXPERIMENTAL PROCEDURES**

**Traditional EB differentiation and research-grade reagents**

Spontaneous EBs formations was achieved by detaching confluent hESCs with EDTA and directly depositing the cell aggregates at a split ratio of 1:1 in a ultralow-adherence plates in 10ml of research-grade KSR composed of Advances DMEM F12, 20% Knockout Serum Replacement, 1% Glutamine (all from Life Technologies) and 0.1mM β-Mercaptoethanol (Sigma). For comparison, GMP Essential 6 media (Life Technologies) was tested. Media was changed every other day for a total of 7 days by collection of the floating EBs to the bottom of a falcon tube for 10 mins followed by re-plating into fresch media. At day 7, EBs were seeded onto a plate prepared by coating with Poly-L-Ornithine solution (P4957-50ml, Sigma) for 30 mins at room temperature, washed twice with DPBS^-/-^ (Life Technolgies) and then coated with 10 μg/ml of laminin (L2020, Sigma) overnight at 4^o^C. Neuronal induction was induced in N2 base media as previously described (Falk et al. 2012).

**Immunostaining**

Cells were fixed for 10 mins with 4% parafolmaldehyde and then washes three times with PBS. For blocking and permeabilization, cells were incubated for 30 mins in PBS plus 10% donkey serum (Serotec) and 0.1% Triton X-100 (Sigma Aldrich). Cells were incubated overnight at 4^o^C with primary antibodies diluted in PBS plus 1% donkey serum and 0.1% Triton X-100. Cells were then washed in 3 times in PBS and incubated for 1hr with fluorescent secondary antibodies (Life Technologies) in 1% donkey serum and 0.1% Triton X-100 (in PBS). Cells were washes 3 times with PBS and counter stained with DAPI (New England Biolabs). Cells were imaged with confocal LSM 700 microscope (Zeiss) and EVOS™ FL microscope (Life Technologies).

Antibodies were against: SOX2 (Bio-techne), Nestin (Abcam), DACH1 (Proteintech), PLZF (Life Technologies), ZO-1 (Bio-techne), GABA (Sigma), TUJ1 ( Biolegend), Tyrosine Hydroilase (Millipore), Nurr1 (Santa Cruz), HB9 (Insight Biotechnology).

**RNA isolation and Q-PCR**

RNA was extracted using the RNeasy kit following manufacturer instructions (Qiagen). An amount of 250 ng of RNA was retro transcribed with the RXN MAXIMA 1^ST^ strand cDNA synthesis kit (Fisher scientific) and diluted 1:40 in ultrapure water. Q-PCR assay was performed with FG, TAQMAN Gex Master Mix (Life Technologies) in a 20 μl reaction including 5 μl of RNA and 1μl of TAQMAN GENE EX Assays. TaqMan assays used were: SOX2 (Hs01053049_s1), PAX6 (Hs00240871_m1), DACH1 (Hs00362088_m1), PLAGL1 (Hs00414677_m1), MMR1 (Hs00201182_m1), NANOG (Hs04260366_g1), PBGD (Hs00609296_g1). Q-PCR was run and analysed with a QuantStudio 12K flex real time machine (Life Technologies) following the TAQMAN comparative program. Samples were normalized to housekeeping gene PBGD.

**Karyology**

NES were prepared for karyotype analysis following methaphase arrest by incubation with 2 μg/ml Colcemid for 4 hr at 37^o^C. Cells were then dissociated in 0.25% trypsin/EDTA (Stem Cell Technologies) and then incubated with hypotonic solution of 0.00375M KCl for 10 mins at room temperature. Cells were centrifuged at 100g for 8 minutes and the pellet suspended drop by drop in fixative solution (3 parts Methanol and 1 part acetic acid; Sigma). Centrifugation and fixation were repeat 3 times before sample were left in 0.5 ml fixative. Cells spreads were controlled prior karyotyping by dropping 10 μl of cell suspension onto a histology slide from a height of approximately 50 cm. Dried samples were stained with DAPI mounting medium (Sigma Aldrich) and harvest of chromosomes clusters confirmed under a fluorescent microscope. Karyotype analysis on fixed samples was performed by Sheffield Diagnostic Genetic Services (Sheffield Children’s Hospital, UK).

**Biostation CT imaging**

Cells undergoing neural induction were imaged at a 2x and 10x magnification with Biostation CT (Nikon) to acquire full-well tiling images every 12 hr for 5 days starting from 1 hr after plating of EBs onto laminin. For proliferation analysis, lt-NES were passaged normally at a ratio of 1:3 into 6-well plates and imaged every 2 hr for 3 days with a 2x2 tiling, 20x magnification. Images were then analysed with CL Quant software following the cell proliferation recipe (Nikon). For terminal differentiation, lt-NES were plated at a density of 40,000 cells/cm^2^ and imaged at 20x magnification every 12 hr for 21 days under spontaneous differentiation protocol. All movies were created with CL Quant (Nikon).
